# Model for Predicting Complications of Hemodialysis Patients Using Data From the Internet of Medical Things and Electronic Medical Records

**DOI:** 10.1109/JTEHM.2023.3234207

**Published:** 2023-01-05

**Authors:** Wen-Huai Hsieh, Cooper Cheng-Yuan Ku, Humble Po-Ching Hwang, Min-Juei Tsai, Zheng-Zhun Chen

**Affiliations:** Department of SurgeryChang-Hua HospitalMinistry of Health and Welfare 103699 Changhua 513007 Taiwan; Institute of Information Management, National Yang Ming Chiao Tung University Hsinchu 300093 Taiwan; Department of NephrologyChang-Hua HospitalMinistry of Health and Welfare 103699 Changhua 513007 Taiwan; Industrial Technology Research Institute 63129 Hsinchu 310401 Taiwa

**Keywords:** Arteriovenous fistula obstruction, electronic medical records, hemodialysis complication prediction, hypotension, Internet of Medical Things

## Abstract

Intelligent models for predicting hemodialysis-related complications, i.e., hypotension and the deterioration of the quality or obstruction of the AV fistula, based on machine learning (ML) methods were established to offer early warnings to medical staff and give them enough time to provide pre-emptive treatment. A novel integration platform collected data from the Internet of Medical Things (IoMT) at a dialysis center and inspection results from electronic medical records (EMR) to train ML algorithms and build models. The selection of the feature parameters was implemented using Pearson’s correlation method. Then, the eXtreme Gradient Boost (XGBoost) algorithm was chosen to create the predictive models and optimize the feature choice. 75% of collected data are used as a training dataset and the other 25% are used as a testing dataset. We adopted the prediction precision and recall rate of hypotension and AV fistula obstruction to measure the effectiveness of the predictive models. These rates were sufficiently high at approximately 71%–90%. In the context of hemodialysis, hypotension and the deterioration of the quality or obstruction of the arteriovenous (AV) fistula affect treatment quality and patient safety and may lead to a poor prognosis. Our prediction models with high accuracies can provide excellent references and signals for clinical healthcare service providers. ***Clinical and Translational Impact Statement***—With the integrated dataset collected from IoMT and EMR, the superior predictive results of our models for complications of hemodialysis patients are demonstrated. We believe, after enough clinical tests are implemented as planned, these models can assist the healthcare team in making appropriate preparations in advance or adjusting the medical procedures to avoid these adverseevents.

## I. Introduction

The prevalence of hemodialysis in Taiwan ranks in the top three globally [1]. The increasing demand for hemodialysis has been caused not only by an aging population but also by the rapid increase of patients with chronic obesity, diabetes, hypertension, and cardiovascular disease (CVD), as well as the progress of diagnostic and therapeutic techniques. According to the Health Promotion Administration statistics, the number of Taiwanese patients who have ever undergone hemodialysis treatment reached 90,000 in 2019 [2]. Because the national health insurance system provides comprehensive medical care [3], the relatively high quality of treatment and low mortality are ensured for most Taiwanese patients with kidney disease [4]. Hemodialysis is a treatment that removes excess toxins and liquid and rebalances blood components. With vascular access, a hemodialysis patient’s blood is led to an artificial kidney with the assistance of a hemodialysis machine, where the blood is exchanged with a special dialysate in excellent artificial fibers by a semipermeable membrane. Then, the unwanted toxins and liquid are removed via the dialysate, and the electrolytes and pH can be adjusted during this process. Because too much water may be drained during the process of hemodialysis and the blood components may change abruptly, adverse reactions such as hypotension and even shock often occur. Therefore, the hemodialysis procedure must be conducted with adjustable parameters such as dehydration rate and dry weight after evaluation by doctors. The treatment process also needs to be closely monitored by nursing professionals to ensure the patient’s safety and health. Moreover, given that hemodialysis involves routine treatment, the arteriovenous (AV) fistula must be repeatedly pricked. The long-term change of AV-fistula-caused pressure difference in blood vessels also often causes vascular intimal hyperplasia and obstruction or increased venous return pressure. These may lead to blood insufficiency. At this time, there is a need to consult with a cardiologist to perform percutaneous transluminal angioplasty to eliminate blocking factors and maintain optimal hemodialysis.

Hypotension and AV fistula events often occur during hemodialysis treatment, and they affect the treatment quality and patient safety and result in a poor prognosis [5]. To predict hypotension and AV fistula obstruction, there is a need to collect large-scale clinical data and perform close observations during the process of hemodialysis. Hemodialysis should be provided to the patients for a sufficient interval [5], but too much dehydrating volume may cause hypotension, which raises a dilemma. Although the obstruction and deteriorated quality of the AV fistula can be judged successfully using photoplethysmography (PPG) or Doppler sonography supported by palpation, this process is laborious and expensive [6], [7].

If artificial intelligence (AI) techniques could be introduced into medical systems, smart predictive models could plausibly provide early warnings of possible future events to medical staff. Based on theoretical considerations, the combination of multimodal data streams should further enhance the prediction results. Therefore, the major advantage of our method is that we established a novel integration platform to collect clinical data coming from the Internet of Medical Things (IoMT) and inspection data stored in electronic medical records (EMR) to establish a so-called human digital twin (HDT) [8]. This HDT not only can predict complications arising from hemodialysis well but also can act as an intelligent assistant to remind the medical team to modify the treatment procedure to avoid a worse outcome. Furthermore, it may provide precision medicine [8] after this prediction model is trained to be a personalized HDT. In this way, the medical team would have enough time to respond to these events or even prevent them, thereby avoiding unnecessary impairments. After reviewing the literature [9], [10] and testing several well-known machine learning (ML) algorithms, i.e., decision tree, random forest (RF), support vector machine (SVM), we adopted the eXtreme Gradient Boosting (XGBoost) algorithm [11], [12] to establish models for predicting hypotension and AV fistula obstruction in hemodialysis patients. Moreover, we chose Pearson’s correlation coefficient (PCC) [13], [14], [15], [16] to determine the linear correlation between variables in this study. In this way, the more critical parameters will be selected beforehand.

Another advantage of the established models is that they are very suitable for further experiments and tests in future clinical research since they demonstrated really good preclinical performance. These models could be monitored by medical professionals in order to measure their effectiveness in future clinical practice. The establishment of a smart and efficient HDT to assist nephrologists would be our long-term goal.

## II. Related Work

### A. Internet of Medical Things

An IoMT system has been used to assess the heart and lung sound quality of neonates [17]. Specifically, Grooby et al. designed a method that can automatically evaluate signal quality to improve the accuracy and reliability of heart rate and breathing rate from noisy neonatal chest sounds using dynamic selection classification. In another study [18], a wearable device with a biosensing facial mask was proposed to detect the pain intensity of a patient using a facial surface electromyogram. The devices were connected to an IoT system for remote pain monitoring. This system can provide an alternative for patients who are unable to self-report. Another wearable continuous-temperature monitor, called Verily Patch, was adopted in [19]. The authors evaluated if it could estimate body temperature precisely and detect fevers early. The experimental results showed that Verily Patch reliably measured body temperatures with an error of 0.35 ± 0.88°F. Chiang et al. [20] investigated the relationships between blood pressure and lifestyle factors using blood pressure monitors and wearable activity trackers. Then the time series data were collected and analyzed by using an ML algorithm. In another study [21], IoMT and deep learning (DL) were combined to propose a system for the remote diagnosis of obstructive sleep apnea (OSA). The physiological signals of human sleep were sent to a cloud server through the IoT, and a DL-based model was used to detect OSA. Elsewhere, Shih et al. [22] retrieved data from the IoMT in a hemodialysis room to establish an early warning system for hemodialysis complications. The predicted symptoms included hypotension, hypertension, and cramp. In this study, it was found that, even though substantial data of IoMT could be recorded, the medical staff did not make regular marks for clinical or uncomfortable events. Therefore, insufficient data were provided to build an early warning system, and the precision of the convolutional neural network learning decreased. It is difficult to confirm the relevance between massive data and target events only using data collected by IoMT, without necessary and appropriate clinical marks generated by healthcare service providers.

### B. Applications of AI in Hemodialysis

Chen et al. [23] used DL to predict the occurrence of hypotension during dialysis, mainly exploring the relationship between hypotension and relevant clinical factors. The results showed that hypotension was positively correlated to body mass index, complications of hypertension, and ultrafiltration volume but negatively related to ultrafiltration rate. In addition, Decaro et al. [24] used ML to predict the blood parameters of dialysis patients, and the visible spectrum parameters of blood were used to train the SVM and artificial neural network model and were then applied to the prediction of hemoglobin (Hb) and oxygen saturation. The study results showed that SVM offered good effectiveness. Moreover, Chiang et al. [25] used the data of a novel PPG and the classification method of SVM to evaluate the quality of dialysis patients’ AV fistula, involving the blood flow volume and degree of stenosis. The experimental data showed that with PPG data, the SVM-based prediction of fistula obstruction could reach a precision of 87.84% and the prediction of blood flow volume could reach a precision of 88.61%. However, as emphasized earlier, the use of PPG or Doppler sonography supported by palpation is laborious and expensive.

With the progression of chronic kidney diseases, the complication of hypertension gradually appears, causing venous incompetence and finally resulting in AV fistula failure. Thus, Bhatia et al. [26] only used data related to the condition of patients’ AV fistula to predict survival using an ML method without using ultrasound. Moreover, with the waveform of arterial blood pressure as input data, Hatib et al. [27] applied an ML algorithm to predict hypotension events because the early changes of the waveform may generate cardiovascular compensatory mechanisms in advance, affecting preload, afterload, and contractility. Gómez-Pulido et al. [28] trained models of ML to predict the possibility of hypotension during hemodialysis. By contrast, Huang et al. [10] further compared the effects of applying different AI prediction models to predict blood pressure during dialysis. The study results showed that RF and XGBoost performed better in the prediction. As end-stage renal disease (ESRD) patients often suffer from CVDs, Mezzatesta et al. [29] adopted an ML method to predict the likelihood of dialysis patients suffering from a CVD. They selected nonlinear SVM, added a radial basis function kernel, supported by a grid search for optimization, and obtained the best effectiveness. The study results also showed sound effects.

Barbieri et al. [30] applied ML techniques to predict the response to anemia treatment. Anemia is one of the most common complications of ESRD patients, and iron and an erythropoiesis-stimulating agent (ESA) are the primary means of treating it. However, the decision on the therapeutic dose is quite difficult and is often averaged. Researchers applied an ML module and confirmed that iron/ESA performed better than traditional methods. The mean absolute error of the hemoglobin generated was predicted to be $\le0.6$ g/dL. In addition, Brier and Gaweda [31] applied AI techniques to ESRD patients to predict changes in hemoglobin by selecting the optimal ESA dose to improve anemia. The AI suggestions showed that the ESA dose could be lowered. Moreover, Makino et al. [32] used a natural language processing technique to analyze patients’ medical data and conduct a timing analysis. They also established diabetic nephropathy (DN) prediction model using ML, with 24 factors being selected. This model achieved high precision of 71% in the successful prediction of DN aggravation, and the DN aggravation group had a significantly high risk of dialysis within 10 years. Furthermore, Goldstein et al. [33] imported five different risk prediction models, which were trained with hemodialysis patients’ electronic health record data to predict the mortality rate. The results showed that a predictive model built with simple methods might not perform worse than those with many complicated methods. Saadat et al. [34] also used a classification tree and a simple Bayes classifier to predict changes in the quality of life of dialysis patients and created an early warning system. Finally, Xiong et al. [35] discussed an important prediction model, which was built based on left ventricular mass (LVM). As the heart would weigh more upon the overhydration of dialysis patients, LVM could be considered a basis for predicting dialysis duration. Even though many research results regarding the application of AI in hemodialysis have been reported, few studies have been performed on the training of ML algorithms with physiological data from the IoMT in the dialysis room and inspection data from the EMR at the same time for predicting hypotension and AV fistula obstruction. We think these predictive models investigated and accomplished by a research team including clinical doctors, ML experts, and IoMT engineers can independently play as smart HDTs to improve clinical utility after sufficient confirmation of their reliability is ensured.

## III. Methods and Procedures

**FIGURE 1.**
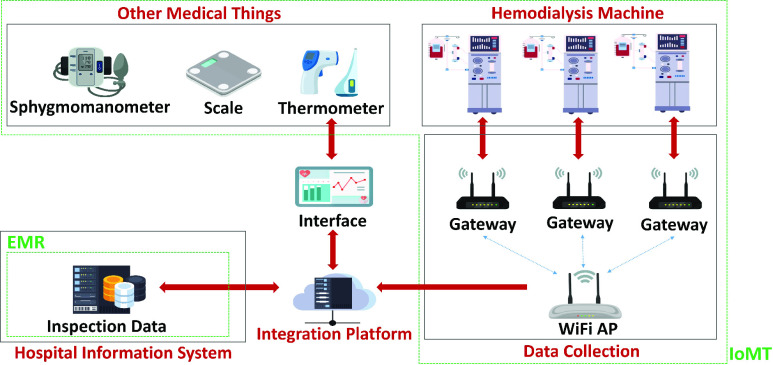
Integration platform of IoMT and EMR.

The IoMT information system was built by the Industrial Technology Research Institute in a dialysis room at Chang-Hua Hospital of the Ministry of Health and Welfare (MOHW) in Taiwan in 2018. Since the system was introduced in 2019, clinical data related to the dialysis process and physiological data from other medical things such as sphygmomanometers, scales, thermometers, etc. of all patients have been collected for quite a long time. Moreover, to improve the effectiveness of predictive models, an integration platform was established to not only store data from the IoMT system but also retrieve individual-case-related inspection data from the EMR as demonstrated in Fig. 1.

This research was approved by the Institutional Review Boards (IRB) of Feng Yuan Hospital of the MOHW under IRB110014. We expected to find the most critical features that may cause the complications of hypotension and AV fistula obstruction in hemodialysis patients from more comprehensively collected data. Accordingly, predictive tools with higher precision and recall rates could be developed to assist therapists and nursing staff in early treatment or prevention. We administered approximately 2,500–2,700 hemodialysis treatments per month. A data subset regarding each dialysis procedure of one patient is considered one data unit. On average, three dialysis procedures should be carried out in one week for each patient and one dialysis procedure should be implemented for around four hours. Thus far, enough units of data have been collected to train and test our predictive algorithms.

Many well-known ML algorithms, such as SVM, decision tree, RF, XGBoost, etc. have been tested, and we adopted the XGBoost algorithm to establish models for predicting hypotension and AV fistula obstruction because of its excellent performance. XGBoost is a widely used ML algorithm, proposed by Chen and Guestrin [11] in 2016. It achieved some impressive feats in many international competitions on ML. Moreover, compared with other learning algorithms, it has the following characteristics and advantages [12]: 1) Owing to the use of parallel processing technology, it can return the output faster than the gradient boosting method does. 2) It can handle and control the problem of overfitting. 3) It provides better learning results on many datasets. 4) It is an algorithm with a decision tree structure. 5) It uses a self-defined loss function to implement classification, regression, and ranking. 6) It can process the situation of sparse data. 7) Through cross-validation, the process of constructing the tree can be stopped in advance when the prediction results are already promising. This can speed up training. 8) Finally, it supports the practice of differentiating sample weights. By adjusting the weights, we can emphasize specific samples.

Moreover, we chose PCC to pick more critical parameters beforehand. Pearson’s correlation is a statistical method used to measure the linear dependence between variables $X$ and $Y$ [13]. Therefore, PCC can describe the degree of correlation between variable $X$ and variable $Y$. On the basis of the well-known definition, the coefficient between two variables $X$ and $Y$ when applied to a population is denoted by $\rho _{XY}$ and can be calculated as follows [13], [14]:

\begin{equation*}\rho _{XY}=\frac {cov(X,Y)}{\sqrt {var(X)} \sqrt {var(Y)}}, \tag{1}\end{equation*}

where $cov(X,Y)$ is the sample covariance of $X$ and $Y$, $\sqrt {var(X)}$ is the sample variance of $X$, and $\sqrt {var(Y)}$ is the sample variance of $Y$. When applied to a sample with paired data $Y\left ({x_{1},y_{1} }\right),\left ({x_{2},y_{2} }\right),\ldots,(x_{n},y_{n})\}$, the coefficient is denoted as follows [13], [14]:

\begin{equation*} \mathrm {r}_{\mathrm {xy}}=\frac {\sum \nolimits _{\mathrm {i=1}}^{\mathrm {n}} {(\mathrm {x}_{\mathrm {i}}-\bar {x})(\mathrm {y}_{\mathrm {i}}-\bar {y})} }{\sqrt {\sum \nolimits _{\mathrm {i=1}}^{\mathrm {n}} {(\mathrm {x}_{\mathrm {i}}-\bar {x})}^{2}} \sqrt {\sum \nolimits _{\mathrm {i=1}}^{\mathrm {n}} {(\mathrm {y}_{\mathrm {i}}-\bar {y})}^{2}}}, \tag{2}\end{equation*}

where $\bar {x}$ and $\bar {y}$ are the sample means.

PCC has the following characteristics [15]:

a) The coefficients for the population and the sample are between −1 and 1. Therefore, the absolute value is ≤1.
b) If all sample data points fall precisely on a straight line, the absolute value of the correlation coefficient is equal to 1.
c) The change in the position and scale of the two variables does not cause the coefficient to change. Therefore, the coefficient is a constant.
d) PCC is symmetric, that is to say, $\rho _{\mathrm {XY}}=\mathrm {\rho }_{\mathrm {YX}}$ and $r_{xy}=r_{yx}$.

We would like to observe and find how a series of time-varying data streams subtly affect the occurrence of complications if we expect to predict them accurately. Therefore, in our model for predicting hypotension, data of dialysis patients around 2-week treatments (i.e., approximately six units of data) were used to predict whether hypotension would occur in the next 2 weeks. In our model for predicting AV fistula obstruction, data of dialysis patients around 3-week treatments (i.e., approximately nine units of data) were used to predict whether AV fistula obstruction would occur in the next 2 weeks. All targets and necessary works can be roughly divided into three stages: (1) integrated data collection and cleanup, (2) selection of critical features, and (3) model buildup and improvement.

**FIGURE 2.**
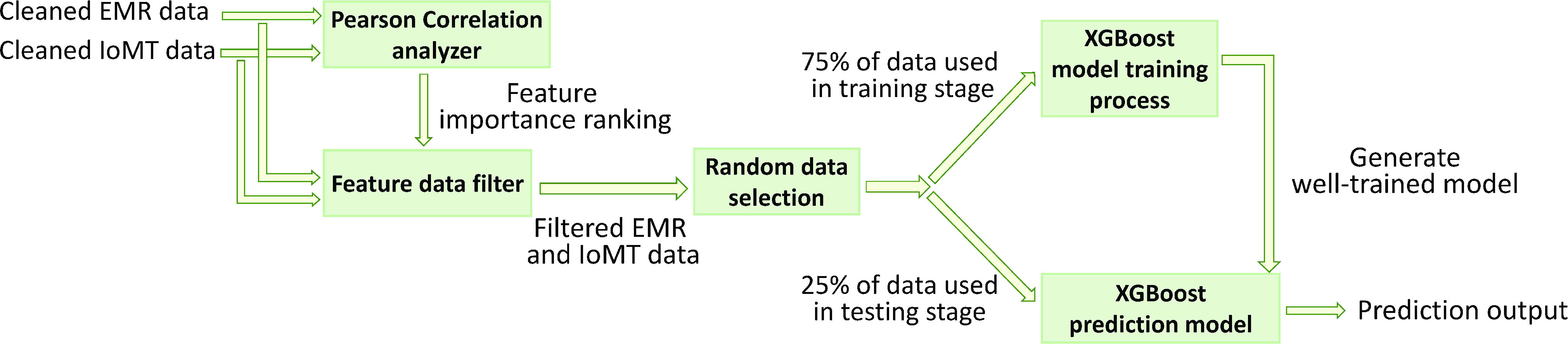
Process of construction and test for predictive models.

(1) Integrated data collection and cleanup: All of the existing inspection data of EMR and physiological parameters of IoMT were collected via the integration platform. As some of the data from other medical things such as sphygmomanometers, scales, and thermometers were manually inputted and marked using mobile devices, there were often missing and abnormal values in the database. Therefore, all of the collected data must be cleaned through our cleaning programs. We delete any records with no value in all columns. If there are missing values in the record, they are filled up with zeros [36], [37]. This is because we do not want to affect the training processes by introducing our estimations. Finally, a complete dataset was organized, as shown on the left of Fig. 2 (cleaned EMR data and cleaned IoMT data).
(2) Selection of critical features: The datasets organized in the first stage were subjected to the PCC method, as shown in Fig. 2, to conduct importance ranking by evaluating the impacts of the features. Then, the less essential factors were removed to reduce disturbance. After further selection based on importance, the remaining features were provided for later training and testing of the predictive models for predicting hypotension and AV fistula obstruction of hemodialysis patients. In this way, we believed that the effectiveness of prediction by our models, such as the precision and recall rates, should be improved.
(3) Model buildup and further improvement: The filtered dataset was randomly divided into a training set with 75% of the data and a testing set with the remaining 25%. The training set was used to train and build the XGBoost models. Our objective was to predict the probability of hypotension and AV fistula obstruction in a time interval of the next 2 weeks. The testing set tested the above predictive models to estimate their precision and recall rates. Some features may be iteratively deleted based on predictive performance. Finally, we considered applying the predictive models built from the actual data of hemodialysis patients to assist clinical staff in improving the outcomes and quality of medical treatments. The detailed modeling and predictive processes are shown in Fig. 2.

## IV. Results

### A. Data Source

To confirm the effectiveness of the complicated models built for predicting hypotension and AV fistula obstruction in hemodialysis patients, we used actual data provided by Chang-Hua Hospital of the MOHW. The IoMT had been used to collect dialysis data of 264 patients from May 2019 to March 2021. This included a total of 52,929 dialysis procedures. The inspection data, doctors’ comments, and digital marks of clinical events that occurred during the dialysis sessions of these 264 patients were also collected from the EMR system. 68 parameters relevant to the complications of dialysis were taken into account. These 68 parameters collected from EMR and IoMT and considered critical for hemodialysis-related complications are listed in Table 1.

**TABLE 1:** Features Collected From emr and iomt

Inspection Data	Hemoglobin	Hematocrit	Platelet	Total protein
	White blood cell	Glutamic oxaloacetic transaminase	Glutamic pyruvic transaminase	Alkaline phosphatase
	Cholesterol	Triglyceride	Creatine	Uric acid
	Glucose	Ca concentration	P concentration	Na concentration
	Cl concentration	K concentration	EMR number	
Before Dialysis	Weight	Blood urea nitrogen before		
During Dialysis 1	Systolic blood pressure	Diastolic blood pressure	Pulse	Blood flow
	Venous pressure	Body temperature	Ultrafiltrate rate	Ultrafiltrate removed
	Dialysis temperature	Final conductivity	Bicarbonate conductivity	
During Dialysis 2	Systolic blood pressure	Diastolic blood pressure	Pulse	Blood flow
	Venous pressure	Body temperature	Ultrafiltrate rate	Ultrafiltrate removed
	Dialysis temperature	Final conductivity	Bicarbonate conductivity	
During Dialysis 3	Systolic blood pressure	Diastolic blood pressure	Pulse	Blood flow
	Venous pressure	Body temperature	Ultrafiltrate rate	Ultrafiltrate removed
	Dialysis temperature	Final conductivity	Bicarbonate conductivity	
During Dialysis 4	Systolic blood pressure	Diastolic blood pressure	Pulse	Blood flow
	Venous pressure	Body temperature	Ultrafiltrate rate	Ultrafiltrate removed
	Dialysis temperature	Final conductivity	Bicarbonate conductivity	
After Dialysis	Weight	Blood urea nitrogen after	Blood urea nitrogen 2-day later	

### B. Prediction of Hypotension

#### 1) Dataset

We used the data of dialysis patients collected within approximately 2 weeks (six hemodialysis treatments because dialysis patients got three a week) to predict the probability of hypotension occurring in the next 2 weeks. All parameters, except the EMR number, were sextupled, so there were a total of $\mathrm {67\times 6+1=403}$ parameters. After data cleaning, preprocessing, and integration had been completed, there were a total of 49,570 units of data in the hypotension prediction dataset. Then, this dataset was randomly divided into a 75% training dataset with 37,177 units and a 25% test dataset with 12,393 units. Moreover, 42,269 units of data belonged to the normal condition, and 7,301 units indicated the occurrence of hypotension.

To evaluate the performance of the predictive models, accuracy (A), complication precision (P), and complication recall rate (R) as described in Eqs. 3–5 were employed. They were defined based on the confusion matrix in Table 2. The accuracy was considered to evaluate the total precision. The reason why we chose complication precision and complication recall rate was based on clinical needs. The healthcare service providers expected the high percentage of positive cases were actual complications so the false alarm rate was low. Therefore, the complication precision should be high and the effort of pre-intervention was not wasted. They also preferred that the high percentage of actual complications were predicted and identified then the missing rate was less as well. Accordingly, the complication recall rate must be good enough and fewer cases missed the preventive treatments.

\begin{align*} \mathrm {Accuracy}&=\frac {TP+TN}{TP+TN+FP+FN}, \tag{3}\\ \mathrm {Precision}&=\frac {TP}{TP+FP}, \tag{4}\\ \mathrm {Recall rate}&=\frac {TP}{TP+FN}. \tag{5}\end{align*}

**TABLE 2:** Confusion Matrix

	Predicted complication	Predicted normal
Actual complication	True positive (TP)	False negative (FN)
Actual normal	False positive (FP)	True negative (TN)

#### 2) PCC for Feature Selection

The original dataset included 403 parameters. We considered that eliminating less critical parameters should reduce interference from them and increase the effectiveness of the XGBoost model. Thus, Pearson’s correlation algorithm was adopted to quantify the relationship between hypotension and these 403 parameters. In general, a larger $r$ value of PCC between hypotension and one specific parameter represents a higher correlation. After much trial and error, the best value of 0.003 was selected. Therefore, 345 parameters with higher importance were chosen for the next stage of XGBoost.

#### 3) XGBoost Model #1

The XGBoost predictive model with 345 input parameters is indexed as model #1. The resulting accuracy was 0.920, the hypotension precision was 0.775, and the hypotension recall rate was 0.644.

#### 4) XGBoost Model #2

The hypotension precision rate of 77.5% for model #1 was acceptable, but its recall rate was only 64.4%, which means that approximately 36% of patients were ignored. We considered that this unsatisfactory result may have been due to substantial interference caused by less critical parameters. Therefore, the parameters with a score lower than 0.0025 based on the feature importance obtained from XGBoost model #1 were dropped, and only the first 161 parameters were used for model #2. After training and testing, the resulting accuracy was 0.926, the hypotension precision was 0.767, and the hypotension recall rate was 0.716.

#### 5) XGBoost Model #3

The hypotension precision rate of 76.7% for model #2 was acceptable, but we still speculated regarding whether its recall rate of 71.6% could be further improved. Therefore, the parameters with a score lower than 0.005 based on the feature importance obtained from XGBoost model #2 were dropped, and only the first 107 parameters were used for model #3. After training and testing works were performed, we obtained a confusion matrix as shown in Table 3.

**TABLE 3:** Confusion Matrix of XGBoost Model #3 for Hypotension

	Predicted hypotension	Predicted normal
Actual hypotension	1430 (TP)	395 (FN)
Actual normal	447 (FP)	10121 (TN)

The resulting accuracy was 0.932, the hypotension precision was 0.762, and the hypotension recall rate was 0.784. The precision rate of model #3 only changed slightly, but the recall rate significantly increased from 71.6% to 78.4%. We thus wanted to build a better model by reducing the number of input parameters to 93. The resulting accuracy was 0.929, the hypotension precision was 0.762, and the hypotension recall rate was 0.751. The precision was almost unchanged, but the recall rate decreased. Furthermore, we analyzed these $\mathrm {107-93=14}$ eliminated parameters and found that some of them are closely related to vascular dynamics, including weight, dehydration, dialysis rate, blood pressure, and pulse. These parameters should not be excluded based on thorough discussions with nephrologists and healthcare service providers. Accordingly, we choose model #3 as a suitable predictive model. Thus, we implemented fourfold cross-validation for model #3. The mean accuracy was 0.932, the mean precision rate was 0.769, and the mean recall rate was 0.766. We considered that our model might also perform well on other datasets based on the abovementioned experimental results.

Based on the feedback collected from nephrological physicians, the performance at this level is good enough to provide clinical assistance. Although there are a few similar research papers predicting hypotension for chronic hemodialysis, they select very different prediction targets and performance indexes. Therefore, it is not easy to compare the prediction efficiency. Ref. [38] built models to predict intradialytic hypotension at the next blood pressure measurement with a sensitivity of 86% and specificity of 81% for both nadir systolic blood pressure < 90 mmHg and < 100 mmHg. Ref. [39] used a recurrent neural network model to predict intradialytic hypotension occurred within 1 hour. This model achieved an area under the receiver operating characteristic curve of 0.94, 0.87, and 0.79 for three different definitions of hypotension respectively.

### C. Prediction of AV Fistula Obstruction

#### 1) Dataset

We attempted to use the data of dialysis patients from approximately 3 weeks (nine hemodialysis treatments, given that dialysis patients got them three times a week) to predict the probability of AV fistula obstruction in the next 2 weeks. One more tag parameter indicating if there is an AV fistula obstruction event or not was added to the dataset for each hemodialysis procedure. All parameters were nonupled, with the exception of the EMR number, giving a total of $\mathrm {68\times 9+1=613}$ parameters. After the data cleaning and integration had been completed, there were 48,902 units of data in the AV fistula obstruction prediction dataset. Then, this AV fistula obstruction prediction dataset was randomly divided into a 75% training dataset with 36,676 units and a 25% test dataset with 12,226 units. Moreover, 47,863 units belonged to the normal condition, and 1,039 units of data indicated AV fistula obstruction. In addition, accuracy, complication precision, and complication recall rate were also used here for the evaluation of performance.

#### 2) PCC for Feature Selection

The original dataset included 613 parameters. We considered that eliminating less critical parameters should reduce the disturbance and increase the effectiveness of the XGBoost predictive model. Thus, Pearson’s correlation algorithm was adopted to quantify the relationship between AV fistula obstruction and these 613 parameters collected in IoMT and EMR. In general, a larger $r$ value of PCC between AV fistula obstruction and one specific parameter represents a higher correlation. After much trial and error, the best value of 0.005 was chosen. Therefore, 269 parameters with higher importance were chosen for the next stage of XGBoost.

#### 3) XGBoost Model

XGBoost with 269 input parameters was selected as the model for predicting AV fistula obstruction. Then, we obtained a confusion matrix, as shown in Table 4. The resulting accuracy was 0.992, the AV fistula obstruction precision was 0.907, and the AV fistula obstruction recall rate was 0.712.

**TABLE 4:** Confusion Matrix of XGBoost Model for AV Fistula Obstruction

	Predicted AV fistula obstruction	Predicted normal
Actual AV fistula obstruction	185 (TP)	75 (FN)
Actual normal	19 (FP)	11947 (TN)

This AV fistula obstruction prediction model had an accuracy rate of more than 99%, a precision rate of approximately 90%, and a recall rate of more than 71%, indicating its outstanding performance in predicting the occurrence of AV fistula obstruction from a clinical perspective. To test the effect of fewer features, we reduced the number of parameters to 225. The resulting accuracy was 99.1%, the AV fistula obstruction precision was 86.7%, and the AV fistula obstruction recall rate was 70.4%. We considered that the precision and recall rates were reduced, so some critical parameters had probably been excluded. Furthermore, we analyzed these $\mathrm {269-225=44}$ eliminated parameters and found that some of them, such as pulse and ultrafiltration rate, are closely related to the vascular condition. These parameters should not be excluded based on thorough discussions with some nephrologists and healthcare service providers. Therefore, we concluded that XGBoost with 269 input parameters is a suitable model for predicting AV fistula obstruction. Next, we implemented fourfold cross-validation. The mean accuracy was 0.992, the mean precision rate was 0.881, and the mean recall rate was 0.702. To the best of our knowledge, less research focuses on predicting the complications related to the AV fistula. Furthermore, nephrological physicians believe that this performance is acceptable based on the clinical point of view.

### D. Discussion

The XGBoost model has been tested many times experimentally. It was found that, while excluding some less important parameters according to the ranking result of the model, the updated XGBoost algorithm for hypotension demonstrated a better recall rate but lower precision. The complication of hypotension is closely related to the dynamics of blood vessels, so these parameters of vascular dynamics are relatively important. Therefore, more parameter data related to vascular dynamics were inputted into the prediction model, and its precision should be higher. Consequently, it is easy to understand why model #1 with 352 parameters had high predictive precision of 77%. However, too many similar features of vascular dynamics may confuse each other for the predictive model, reducing the recall rate. It is not that easy to distinguish potential hypotension from normal status. Therefore, the recall rate significantly increased for predictive models with fewer features, but the precision decreased. We found that most of the excluded parameters are related to vascular dynamics, so its precision would naturally decrease. However, the reason for this may be that so many essential parameters related to vascular dynamics are deleted and the inspection data and medical record data are left. We think that this situation is due to the field design of the original dataset. The data from EMR were only provided once or several times every month, but the vascular dynamics data from IoMT were collected many times during each dialysis procedure. Because of this significant disparity in quantity, it is possible that the essential parameters of vascular dynamics were diluted and finally ranked with lower importance. Then, they were often excluded during parameter selection based on a specific threshold score. However, it is not acceptable to exclude too many vascular dynamic parameters with predictive function because they are major factors in blood pressure based on consideration of the physiology theory. This is why we chose model #3 as our suitable predictive scheme. For the complication of AV fistula obstruction, PCC generated a good set of features. Then, no more selection steps were necessary.

In the process of hemodialysis treatment, a large amount of blood leaves the human body and enters the dialysis system, which naturally contains a large number of risks. Mild hypotension cases may cause discomforts such as cramps, dizziness, or vomiting. In severe cases, fainting, irregular heart rate, or sudden death could happen. Once the vascular channel used in dialysis is blocked, infected, or bleeding, it may result in the extra use of medicine, invasive treatment, or even hospitalization and surgery. To avoid these adverse dialysis complications, it depends on intensive patient monitoring. However, this consumes a lot of time and energy of healthcare service providers. If the IoMTs are used to collect data from the dialysis machines, sphygmomanometer, scale, thermometer, etc. and the inspection data from EMR are integrated to build a more comprehensive dataset, then we think the excellent predictive models for the above complications can be built. As long as the models’ accuracies and reliabilities are sufficient, they may play as faithful HDTs in clinical treatment to greatly reduce the risk of patients during dialysis, improve the quality of treatment, and lower the burden on the medical staff. This can also promote the future development of HDTs and precision medicine.

## V. Conclusion

Most patients undergoing dialysis procedures have chronic kidney disease, so long-term dialysis is required. Therefore, there is a high risk of suffering from complications during treatment. Hypotension and the deterioration of the quality or obstruction of the AV fistula are diagnostic and treatment problems for dialysis patients. After the diagnosis is confirmed, it can be well dealt with according to the current medical capability in Taiwan. However, for healthcare service providers on the dialysis team, the most critical issue is predicting the likelihood of these complications in dialysis patients in the short term. Then, the medical team has enough time to modify the treatment procedure to avoid the worse outcome. Owing to the abovementioned factors, we collected many parameters of dialysis patients from the IoMT and EMR. We then built predictive models to predict the probabilities of complications for dialysis patients. We found correlations between the collected parameters and complication themes by learning from statistical principles, ML algorithms, and subtle changes among these appropriate parameters. From the experimental results regarding data from Chang-Hua Hospital of the MOHW, we could use the essential parameters collected during dialysis procedures and inspection data in the past few weeks to successfully predict the possibility of hypotension and AV fistula obstruction in the next 2 weeks. The precision and recall rates reached approximately 71%–90%, which are good enough to act as clinical references. It also means that the false omission rate of our model is low. Suppose that we predict that a dialysis patient will have complications with a high enough precision during the next 2 weeks. In that case, the healthcare team can make appropriate preparations for these complications in advance or modify the medical procedures to avoid complications. This is much better than providing patients with the diagnosis and treatment every time a complication occurs.

The prediction period for hemodialysis complications is selected as 2 weeks. Also, the threshold of hypotension is fixed in all experiments. These two limitations of our research could be released in the future. We think the prediction of hemodialysis complications with high precision for the next dialysis procedure is more helpful to the healthcare team and the threshold of hypotension should be decided according to the unique constitution of each patient.

Although the inspection data of EMR have been added for prediction, there should be more related factors causing the occurrence of hypotension and AV fistula obstruction, such as other diseases of the patient, medicine in use, nutritional status, etc. The follow-up research may consider adding more personalized medical information so as to increase prediction accuracy. In addition, the dialysis procedure is not performed every day, the physiological parameters during the dialysis process are not measured continuously and the actual adverse events may not occur during the dialysis process. To solve these problems, the wearable measuring instrument for vital signs could be a good choice to collect more patients’ data. Although the accuracies, precisions, and recall rates of our models have reached a superior level, they are still in the stage of data validation. In the near future, we expect to carry out pre-intervention in the clinical treatment according to its early warning information, such as performing angiography or adjusting medical prescriptions. Then we may randomly assign dialysis cases to the experimental group and control group individually to observe whether the predictive models can reduce the occurrence of hypotension and AV fistula obstruction in the dialysis room.
